# Prophylactic measures for reducing deep vein thrombosis risk in post-operative critical care patients

**DOI:** 10.6026/973206300213554

**Published:** 2025-10-31

**Authors:** Robin Abraham, Mahalakshmi B, Siva Subramanian N

**Affiliations:** 1 Department of Medical & surgical Nursing, Nootan College of Nursing, Sankalchand Patel University, Visnagar, Gujarat - 384315; India; 2 Department of Pediatric Nursing, Nootan College of Nursing, Sankalchand Patel University, Visnagar, Gujarat - 384315, India; 3 Department of Psychiatric Nursing, Nootan College of Nursing, Sankalchand Patel University, Visnagar, Gujarat - 384315, India

**Keywords:** Deep vein thrombosis (DVT), post-operative patients, critical care units, knee-length compression stockings, leg exercises

## Abstract

The effect of knee-length DVT stockings and structured leg exercises in reducing the risk of deep vein thrombosis (DVT) is of interest. Participants were randomly divided into experimental (n=60) and control (n=60) groups and DVT risk was assessed using the Modified Caprini Risk Score at baseline and Wells Criteria post-intervention. Baseline scores were comparable, confirming group homogeneity, but post-intervention analysis showed a significant reduction in DVT probability in the experimental group. The mean Wells score was 0.82 for the experimental group compared to 5.37 in the control group, with p < 0.001 indicating a highly significant difference. Data shows that simple, non-pharmacological interventions can effectively lower DVT risk in high-risk surgical patients and should be incorporated into routine post-operative care to reduce thromboembolic complications.

## Background:

Deep vein thrombosis (DVT) is a serious and potentially life-threatening condition that commonly affects post-operative patients, particularly those admitted to critical care units. It is characterized by the formation of a blood clot in a deep vein, usually in the lower extremities and can lead to complications such as pulmonary embolism (PE), chronic venous insufficiency and post-thrombotic syndrome [1]. Among hospitalized patients, surgical intervention, prolonged immobility and multiple comorbidities contribute significantly to an increased risk of venous thromboembolism (VTE), which includes both DVT and PE [2]. Globally, the incidence of hospital-acquired DVT remains high, despite the availability of effective prophylactic strategies [3]. In India, where the burden of surgical admissions is rapidly increasing, particularly in urban tertiary care centers, post-operative DVT is an under-recognized threat due to limited awareness, underutilization of assessment tools and inconsistent application of preventive measures [4]. Many post-operative patients admitted to intensive care units (ICUs) experience reduced mobility, fluid imbalances and tissue trauma-all of which contribute to the triad of hypercoagulability, venous stasis and endothelial injury as described in Virchow's triad, the foundational concept for thrombus formation [5]. The current standard of care for DVT prevention includes pharmacological interventions such as low molecular weight heparin and mechanical methods such as graduated compression stockings, intermittent pneumatic compression devices and early ambulation or limb exercises [6]. The Caprini Risk Assessment Model is one of the most widely used and validated tools for estimating DVT risk in surgical patients [7]. It takes into account multiple patient factors such as age, BMI, comorbidities, surgical duration and mobility status. The Wells Criteria is useful for evaluating the clinical probability of DVT based on patient symptoms and signs [8]. Therefore, it is of interest to describe the effectiveness of knee-length compression stockings and structured leg exercises in reducing the risk of DVT among post-operative patients in critical care units.

## Methodology:

## Research design and approach:

A quantitative research approach with a true experimental post-test only control group design was used in this study.

## Study setting and population:

The study was conducted in the critical care units of two tertiary care hospitals in Bharuch, Gujarat: Welfare Hospital and Research Centre and Kaka Ba Hospital. The target population consisted of post-operative patients admitted to critical care units following elective or emergency surgeries.

## Sampling technique and sample size:

A total of 120 post-operative patients were selected through convenience sampling and were then randomly assigned into two equal groups: experimental (n=60) and control (n=60).

## Intervention and tools:

The experimental group received knee-length DVT stockings and leg exercises (3 times daily for 4 days), while the control group received routine care. Data were collected using the Modified Caprini Risk Assessment Scale (for baseline risk) and Modified Wells Criteria (for post-test assessment).

## Data analysis:

Data were analyzed using descriptive statistics (mean, SD, frequency) and inferential statistics. An independent t-test was used to compare post-test scores and p < 0.05 was considered statistically significant.

## Results:

Table 1 (see PDF) shows that most post-operative patients in both groups were aged 41-60 years, male, unemployed, had a healthy BMI and underwent elective surgery lasting 3-4 hours. Slightly limited mobility was most common, hypertension was the leading co-morbidity and very few had a previous history of DVT. Table 2 (see PDF) indicates that pre-test mean DVT risk scores were similar between the experimental and control groups (p = 0.861). Post-test results revealed a marked reduction in the experimental group (0.82) compared to the control group (5.37), demonstrating a highly significant effect of the intervention (p < 0.001). Figure 1 (see PDF) shows mean pre- and post-test scores for experimental and control groups in reducing DVT risk among post-operative patients, highlighting a greater improvement in the experimental group after intervention.

## Discussion:

This study shows that mechanical prophylaxis effectively reduces DVT risk in post-operative patients. Post-test Wells scores were significantly lower in the experimental group compared to controls (0.82 vs. 5.37; p < 0.001). Similar findings are reported in previous studies. A meta-analysis showed that graduated compression stockings (GCS) significantly lower DVT incidence in surgical patients [9]. A randomized trial demonstrated up to 50% reduction in thrombotic events with stockings alone or combined with drugs [10]. The Caprini Risk Assessment Score, used for risk stratification, is validated across surgical populations [11]. In our study, baseline Caprini scores were similar, but only the experimental group showed post-test improvement. Evidence from abdominal, orthopedic, cardiac and cancer surgeries confirms that intermittent pneumatic compression (IPC) and GCS reduce venous stasis and improve calf muscle pump function [12]. In lung cancer surgery, intraoperative IPC significantly reduced DVT [13]. Combining mechanical methods with early ambulation is safe, cost-effective and preferred when anticoagulation is contraindicated [14, 15- 16]. Cochrane reviews recommend GCS and IPC for immobile or high-risk surgical patients [17]. The American College of Chest Physicians also supports mechanical prophylaxis for moderate-to high-risk patients when pharmacologic options are unsuitable [18]. Further data show additive benefit when GCS are combined with anticoagulation, reducing VTE more than anticoagulation alone [19]. Lippi *et al.* (2011) reported mechanical compression lowers VTE risk by two-thirds and by half when combined with drugs, without raising bleeding risk [20]. The CLOTS 3 trial confirmed IPC reduces proximal DVT in immobile stroke patients [21]. Collectively, these findings support mechanical prophylaxis-alone or with drugs-as an effective, safe strategy for preventing VTE.

Multiple studies emphasize the effectiveness of mechanical prophylaxis-such as graduated compression stockings (GCS) and intermittent pneumatic compression (IPC)-as low-cost, low-risk strategies, especially critical in resource-limited settings with bleeding risks or limited pharmacologic access. A systematic review concluded that IPC may be superior to GCS alone and combining IPC with GCS may be more effective for high-risk surgical patients [12]. Another meta-analysis found that IPC offers comparable protection to anticoagulants for venous thromboembolism (VTE) prevention, with potentially lower bleeding risk when used instead of pharmacologic [22]. In cases where pharmacologic prophylaxis is contraindicated-such as in spinal cord injury or high bleeding risk scenarios-mechanical methods are endorsed as a safe alternative [23]. In Asian populations, mechanical VTE prophylaxis is recommended when pharmacologic options are limited, aligning with Asia-Pacific surgical consensus [24]. Furthermore, using risk stratification tools like Caprini can facilitate proactive identification of high-risk patients for mechanical prophylaxis [22, 23]. Our study builds on this evidence, showing that simple interventions-leg exercises and stockings-applied systematically in postoperative care significantly reduced post-test Wells scores. Despite limitations like its single-center design, short-term outcome focus and lack of adherence measurement, the findings affirm the potential of mechanical methods to prevent complications, improve outcomes and reduce hospital stays in settings with constrained resources. Future research should include multicenter trials, comparisons with pharmacologic approaches and cost-benefit analyses to inform national guidelines in LMICs like India. Additionally, it did not compare mechanical prophylaxis with pharmacological methods and patient adherence to exercises was assumed rather than measured. Future research should involve multicenter studies, evaluate both mechanical and pharmacological strategies and conduct cost-benefit analyses to inform national guidelines, particularly in low- and middle-income countries like India. Despite these limitations, mechanical prophylaxis remains a safe, accessible and essential approach for reducing post-operative DVT risk in critical care.
